# Reduced visual attention in heterogeneous textures is reflected in occipital alpha and theta band activity

**DOI:** 10.1371/journal.pone.0187763

**Published:** 2017-12-07

**Authors:** Tobias Feldmann-Wüstefeld, Makoto Miyakoshi, Marco Alessandro Petilli, Anna Schubö, Scott Makeig

**Affiliations:** 1 Department of Psychology, University of Chicago, Chicago, IL, United States of America; 2 Swartz Center for Computational Neuroscience, Institute for Neural Computation, University of California San Diego, San Diego, CA, United States of America; 3 Department of Psychology, University of Milan-Bicocca, Milan, Italy; 4 Faculty of Psychology, Experimental and Biological Psychology, Philipps-University Marburg, Marburg, Germany; Harvard Medical School, UNITED STATES

## Abstract

Increasing context heterogeneity has been found to reduce attention deployment towards an embedded target item. Heterogeneity in visual search tasks is typically induced by segmenting the background into several perceptual groups. In the present study, however, context heterogeneity was induced by varying whole-field heterogeneity, i.e., the degree of distractor variability within the entire context. This allowed us to (i) more gradually vary context heterogeneity, and (ii) investigate attention deployment on a whole-field scale. Results showed that both search performance and amplitude of the N2pc (lateralized ERP; posterior contralateral negativity in the N2 range) monotonically decreased with increasing context heterogeneity, which confirmed that there was less efficient attention deployment for more heterogeneous contexts. The amplitude of the bilateral N2 exhibited a U-shaped function, suggesting global perception for the lowest and highest levels of heterogeneity, but local processing for intermediate heterogeneity levels. Independent component analyses revealed an occipital ERP-contributing effective source cluster that may reflect stimulus representations on a saliency map. With increasing heterogeneity, these sources exhibited more theta band activity for distractors and less theta band activity for targets. Alpha band activity of a second component cluster varied with heterogeneity level, and low-theta/delta activity of a third source cluster distinguished target presence versus absence. In sum, our results suggest that independent brain sources contributed to the differential processing of heterogeneous versus homogeneous contexts.

## Introduction

Visual attention allows us to actively select the most relevant visual information from the vast amount of information that we are confronted with at every glance. As a result, relatively little information enters our visual short-term memory, which allows information overload to be averted [1,2]. The visual search paradigm mimics the need of selective attention in everyday situations by presenting a relevant item (target) among irrelevant items (distractors) and letting observers search for the target. Although distractors are by definition task-irrelevant, their properties can strongly influence attention deployment in the visual field. Distractor heterogeneity can affect pre-attentive processing [3–5], target selection [6–11], suppression of salient distractors [12] and long-term memory encoding [13].

In a seminal study by Duncan & Humphreys [3], participants had to detect a T letter serving as the target item among L letters serving as distractor items. Context heterogeneity was manipulated by using either Ls of only one orientation (non-heterogeneous) or Ls of four different orientations (heterogeneous). Detection of targets in non-heterogeneous contexts was found to be faster than in heterogeneous contexts. The authors argued that the advantage of non-heterogeneous contexts was due to differences in how the context was processed: When all distractors were identical (non-heterogeneous context), they could be grouped into one perceptual unit, and the decision of whether a target was present or not could be made on a global level. Any odd element in the visual field would indicate target presence, whereas the lack thereof would indicate target absence. When distractors were heterogeneous, no single item pops out, and, thus, the decision about target presence has to be made on a local level. To follow up on this, Schubö et al. [11] varied the number of perceptual groups within the visual field systematically. Observers had to detect a single oblique line serving as the target within large contexts of horizontal and vertical lines. Although the same two distractor types were used in all three conditions, they were arranged to induce different levels of heterogeneity (*arrangement heterogeneity*). Contexts were arranged to either show no heterogeneity (e.g., horizontal lines only), intermediate heterogeneity (two smaller perceptual groups of identical orientation within each group, e.g., the left half comprised of horizontal and the right half comprised of vertical lines), or strong heterogeneity (horizontal and vertical lines were randomly arranged throughout the entire context). Response times and false responses increased with increasing heterogeneity. Other studies that varied arrangement heterogeneity found that the N2pc, a marker of visual attention [14,15], was more pronounced for targets in less heterogeneous contexts, suggesting more efficient attention deployment to targets if fewer perceptual groups have to be examined for target presence [16,17].

A neural marker of global processing is the posterior N2 component. When contexts foster more global processing, because they show no heterogeneity, the N2 amplitude of the stimulus-evoked ERP over posterior scalp increases. This is particularly true in target-absent compared to target-present trials, but this is not the case for contexts that had to be processed more locally [9,11,18]. Similarly, N2 amplitude is larger when observers have to attend global features in Navon figures than when they have to attend local features [19]. In the present study, we will use the differential N2 (target absent–target present) as a marker of global vs. local processing in contexts of varying heterogeneity.

There are at least two ways of inducing varying levels of heterogeneity: *arrangement heterogeneity* and *whole-field heterogeneity*. As described above, context heterogeneity has often been operationalized in terms *arrangement heterogeneity*, i.e., the number of distractor identities is kept constant but is differently arranged [11,18,20–22]. However, search performance can also depend on *whole-field heterogeneity*, i.e. similarity among distractors [23–26]. For example, Rosenholtz [26] presented target horizontal line elements (0°) among identically oriented distractors at 30° (the no-heterogeneity condition), among distractors at 30° and 50° (semi-heterogeneous condition), or among distractors at 30°, 50° and 70° (heterogeneous condition). Accuracy decreased with increasing *whole field heterogeneity*. The limiting factor in attention deployment was not the number of perceptual groups. In this case, the distractors were randomly distributed in the search array, which created clear borders between predefined regions in the visual field. Rather, the availability of salience signals was the limiting factor in attention deployment: Displays with high distractor orientation variability showed smaller local contrasts between targets and distractors. That is, the signal-to-noise ratio on the salience map was decreased compared to displays with low variability, and the representation of the target stuck out less. This makes target detection more error-prone and/or slower, causing performance to deteriorate. The present study sought to investigate whether the differential search performance observed for different levels of whole-field heterogeneity is due to differential attention deployment.

How are grouping processes affected by the whole-field heterogeneity that we will use in the present study? When distractors are randomly distributed in a search array, the number of perceptual groups should not depend on clear borders between predefined regions in the visual field (e.g. hemifields or quadrants). Instead, they should be dependent on inherent properties of the more or less variable arrangement of stimuli. When distractors are identical (no heterogeneity), contexts should be grouped on a global level. With increasing heterogeneity, nearby cluster of distractors may be grouped because of local similarity and because of sufficient local contrast that allows borders between such groups to emerge. Although more variation fosters local borders that demarcate clusters, it also undermines local grouping, counteracting clustering (see Nothdurft, 1992 or Feldmann-Wüstefeld & Schubö, 2013 for systematic variation of local heterogeneity and border contrasts). However, it is unclear whether some level of heterogeneity on the local level can lead, again, to global processing.

### Relationships between EEG oscillations and visual attention

For many years, event-related potential (ERP) measures played an important role in studies of visual attention [27,14,28]. More recently, richer computational methods like event-related spectral shifts or perturbations in different frequency bands have made it possible to look into EEG data more thoroughly [29]. These are extremely useful tools for applying inverted encoding models [30], and for calculating EEG measures for statistically independent EEG sources separated from the channel data, rather than for individual scalp channels [31]. Recent research suggests that the alpha, and perhaps other frequency bands, is closely related to the deployment of visual processing resources, i.e. their relative strengths affect which percepts are further processed and which are filtered out. For example, covert attention shifts are associated with alpha activity ‘blocking’ or ‘flooding’ [32,33] and ‘nested’ phase-amplitude coupled theta/alpha and gamma oscillations may play a crucial role in sorting unattended visual items in order of relevance to optimize shifts of attention [34].

Previous results suggest that targets in non-heterogeneous contexts are more salient than in heterogeneous contexts and that different modes of processing may be involved in processing non-heterogeneous contexts (strong grouping allowing for more global processing) versus heterogeneous contexts (involving more time-demanding local processing). Therefore, we hypothesize that these task conditions may also have different EEG correlates. The ERP components may be difficult to disentangle because the ERP components associated with this kind of visual search task (N2pc, bilateral N2) are most pronounced at approximately the same scalp locations (posterior-occipital) and scalp EEG data channels themselves have poor spatial resolution, mechanisms that reflect (i) the availability of saliency signals, (ii) differences in target-present vs. target-absent trials and (iii) those involved in processing contexts of varying heterogeneity may be spatially heavily overlapping. Statistically independent cortical EEG source processes involved in processing contexts of low or high heterogeneity can be retrieved by decomposing the scalp EEG data with independent component analysis (ICA) [31,35–37]. Thus, we proposed to test whether power in specific frequency bands is differentially affected by visual search for targets in contexts of varying heterogeneity, and to determine in which areas of visual cortex these effects originate.

### Rationale of using ICA

To analyze EEG data, we used independent component analysis (ICA). It was proposed that the spatial filter ICA explains the process of volume conduction and scalp mixing of cortically generated source EEGs [38]. This claim has been repeatedly demonstrated [37,39]. The critical assumptions of ICA are, 1) source EEG signals be mixed linearly and instantaneously; 2) spatial locations of EEG sources against scalp channels are fixed throughout the recording time (spatial stationarity); 3) EEG signals are stationary i.e. have similar variance throughout the recording time (temporal stationarity) 4) EEG signals/artifacts have non-Gaussian distributions. In practice, these assumptions mostly hold. The theoretical possibility of why ICA should work with scalp-recorded EEG data can be explained as follows: when volume conductance is seen from sources, it is obvious that scalp channels receive mixed signals from all sources in addition to non-brain artifacts. The mixed signals are redundant, which also means their distributions are more Gaussian due to central limit theorem. Assuming this Gaussianity arises only from mixing and the mixing process itself is uninteresting, the signal of interest should have a distribution of minimized non-Gaussianity [40]. Because ICA maximizes non-Gaussianity of the decomposed signal distribution, it un-mixes the scalp signals and recovers source signals.

### Rationale of the present study

The present study sought to investigate attention deployment in large arrays (144 stimuli per display) of varying whole-field heterogeneity. We gradually varied the orientation contrast of simple line elements (serving as distractors) encompassing the entire visual field. There were four heterogeneity conditions: the distractors’ orientation did not vary at all (least heterogeneous condition), varied within a range of 12°, a range of 24° or a range of 36° (most heterogeneous condition). In 50% of the trials a target (orthogonal to the mean orientation of the distractors) was present. We expect to find that (i) search performance (accuracy, d-prime) decreases as a function of heterogeneity, (ii) these search performance differences are due to differential attention deployment, (i.e. the N2pc decreases as a function of heterogeneity) and (iii) the differential bilateral N2 decreases with increasing heterogeneity due to less global processing. (iv) In addition to that, we expect to find measures of maximally independent processes that index differential processing of heterogeneity and/or differential processing of target presence or absence with an independent component analysis (ICA) methodology. This may enable us to look at the cortical EEG dynamics supporting differential availability of saliency signals and different search modes in high and low heterogeneity conditions.

## Materials and method

### Participants

22 paid volunteers (9 male) aged 21–34 years (M = 25.2, SD = 3.6) participated in the experiment. All but one were right-handed and all had normal or corrected-to-normal vision. The experiment was conducted with the written informed understanding and consent of each participant. The experiment has been approved by the research ethics committee of the Philipps-University of Marburg. One additional participant had to be excluded from data analysis due to extensive artifacts in the data (see below for criteria).

### Stimuli and apparatus

**Fig 1:**
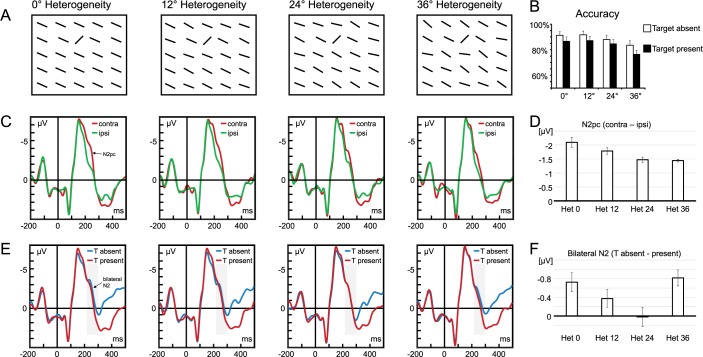
Stimuli, behavioral, and ERP results. (A) In the 0° heterogeneity condition, all distractor lines were identical. In the 12°, 24° and 36° heterogeneity condition, orientation of each distractor varied randomly within 12°, 24°, or 36°. Target orientation was always in 69° contrast to average distractor orientation. (B) Mean accuracy for each of the four distractor orientations. (C) Lateralized ERP. Average ERP waveforms recorded at a posterior-occipital electrode pool (PO7, PO8), evoked by search display presentations, separately for sites contra- and ipsilateral to the target. The difference between contralateral (red lines) and ipsilateral (green lines) activity constitutes the N2pc. (D) Mean N2pc amplitude. Difference between contra- and ipsilateral activity at PO7/PO8, separately for the four heterogeneity conditions. (E) Bilateral ERP. Average ERPs recorded at a posterior-occipital electrode pool (PO7, PO8), evoked by search display presentations. Results are shown separately for the four distractor heterogeneity levels. Blue lines represent the ERP time courses in target-absent trials; red lines the ERP time courses in target-present trials. The difference between blue and red lines constitute the differential bilateral N2. (F) Differential bilateral N2. Difference between N2 for target-absent and target-present trials. Grey shaded rectangles show the time window (220–300 ms) for statistical analyses. Error bars in B-F represent standard errors of the mean, corrected for individual differences [66]. Note that the deflection in the ERP in the baseline period is due to the onset asynchrony of fixation cross and search display (300 ms).

Participants were seated in a comfortable chair in a dimly lit, sound attenuated chamber, with a gamepad in their hands. Participants had to use their left and right index finger to press two buttons on the back of the gamepad. Stimulus presentation and response collection were controlled by a Windows PC using E-Prime routines. All stimuli were presented on a 19-inch computer screen with a 100-Hz refresh rate, placed at a distance of 100 cm from the observers. A grey hue (RGB: 211, 211, 211) served as the background color for all displays. Search displays contained a matrix of 12 x 12 black lines. Single lines had a length of 1.1° of visual angle, the matrix had a diameter of 14.2°. There were four heterogeneity condition: in the heterogeneity-0 condition, all lines were rotated 24° (clockwise from a horizontal line); in the heterogeneity-12 condition, lines varied randomly within a range of 12° (18° - 30°; mean orientation 24°); in the heterogeneity-24 condition, lines varied randomly within a range of 24° (12° - 36°; mean orientation 24°) and in the heterogeneity-36 condition, lines varied randomly within a range of 36° (6° - 42°; mean orientation 24°); cf. the examples in Fig 1A.

In target present trials, one of the lines served as the target and was rotated -45° (i.e. 69° contrast to the mean line orientation in all four heterogeneity conditions). The target appeared with equal probability at one of four locations replacing a distractor, at the central location of the four imaginary quadrants at an eccentricity of 4.1° from screen center. Target-present and target-absent trials were equally likely. The mask consisted of a 12 x 12 array of asterisk-like elements constructed by superimposing three randomly rotated black lines in each matrix cell.

### Procedure

Each trial began with the presentation of a central fixation dot (2x2 pixels) that remained on the screen throughout the entire trial. After 300 ms, the search display was presented for 100 ms and was then masked with asterisks. At 800 ms, the mask disappeared and a blank screen with a fixation dot was shown until participants responded (if they had not already responded during mask presentation). Participants were asked to indicate the presence or absence of a target by pressing the respective button on the back of the gamepad using their left or right index finger; button assignment was balanced across participants. Participants could respond as soon as the search display appeared on the screen. Response accuracy, but not response speed, was stressed in participant instructions and there was no response time limit. After the participant response, a blank screen was maintained for 600–800 ms until presentation of the fixation point indicated the beginning of the next trial. The experiment consisted of 800 trials, 100 trials in each of the eight conditions (four heterogeneity conditions by target presence/absence). All conditions were randomly mixed throughout 16 blocks (50 trials per block). After each block, performance feedback (percent accuracy of target detection) was given to participants. The 16 experiment blocks were preceded by one block of 50 practice trials.

### EEG recording

EEG was recorded from 64 Ag–AgCl electrodes (according to the International 10–10 System) using a BrainProducts (Gilching, Germany) *actiCap* system. Horizontal and vertical EOGs were recorded bipolarly from the outer canthi of the eyes and from above and below the observer’s left eye, respectively. All electrodes were referenced to Cz and re-referenced off-line to the average of all electrodes. Electrode impedances were kept below 5 kΩ. Sampling rate of the Brain Products (Gilching, Germany) EEG recorder was 1000 Hz with a high cutoff filter of 250 Hz and a low cutoff filter of 0.1 Hz.

### Data analysis

#### Behavioral data

Mean accuracy and reaction times (RT) were calculated for each participant, separately for each heterogeneity condition (0°, 12°, 24°, 36°) and each trial type (target-absent, target-present) and submitted to a 4×2 ANOVA. Trials with false responses were removed from the RT analysis. Trials with exceedingly long RT (+/- 2 SD from mean RT calculated separately for each participant) were removed from accuracy and RT analyses. In addition, signal detection measures (d-prime as a measure for target detection sensitivity, natural logarithm of beta (ln(β)) as a response criterion measure) were calculated from accuracy. Mean accuracy and RT were entered into a two-way repeated measures analysis of variance (ANOVA) with factors Trial type (target-absent vs. target-present) and Heterogeneity (0° vs. 2° vs. 24° vs. 36°) and mean d-prime and ln(β) were forwarded to a one-way repeated measures ANOVA with the factor Heterogeneity (0° vs. 2° vs. 24° vs. 36°). For these and all other ANOVAs described in this article, Greenhouse-Geisser correction was applied when necessary.

#### ERP data

EEG data were averaged off-line within 700-ms epochs time-locked to the search display onset and including a 200-ms pre-stimulus baseline. Only trials associated with correct responses were analyzed. Trials in which the vertical EOG channel revealed blinks (indicated by any absolute voltage difference in a segment exceeding ±60 μV) or the horizontal EOG channel revealed eye movements (step criterion; average voltage difference between first and second half of a 200 ms time window moving in 50 ms steps exceeding 15 μV indicated an eye movement) were excluded from further analysis. To avoid false alarms due to noise in the artifact rejection procedure, EOG channels were filtered offline with a 25 Hz lowpass filter. Data from one participant had to be excluded due to extensive artifacts (initial data loss of 65.4%). Across the remaining 22 participants, 15.0% of the trials showed contamination with eye movements or blinks (SD = 12.6%). Trials for individual scalp channels were excluded from further analysis when the absolute voltage in a segment exceeded ±30 μV.

To analyze the N2pc amplitude, for each participant and level of heterogeneity, EEG epochs centered on presentations of arrays containing targets were averaged at two parietal electrode (PO7 and PO8) sites contralateral and ipsilateral to the target. To obtain the mean amplitude, a data-driven approach was chosen. For each participant, we used those time points within 150–350 ms for averaging for which the difference between the ERP contra- and ipsilateral to the target was negative. Mean lateralized amplitude was then forwarded to a one-way repeated measures ANOVA with the factor Heterogeneity (0° vs. 2° vs. 24° vs. 36°) for statistical analyses.

For (bilateral) N2 analyses, target-absent and target-present trials were averaged for each participant and level of heterogeneity. For the bilateral N2, mean amplitude was computed for the channel mean of PO7 and PO8 in latency windows 220 ms to 300 ms, the same time window as in a previous study [41]. Resulting amplitudes were entered into a two-way repeated measures analysis of variance (ANOVA) with factors Trial type (target-absent vs. target-present) and Heterogeneity (0° vs. 2° vs. 24° vs. 36°).

#### Independent component analysis (ICA)

*Preprocessing*. Data were imported to EEGLAB version 13.6.5 [31] running under Matlab (The MathWorks, Natick, MA) version 2013a. An FIR high-pass filter (Hamming windowed, transition bandwidth 1 Hz) was applied with cutoff frequency 0.5 Hz. Continuous data were cleaned using EEGLAB plug-in *clean_rawdata* including artifact subspace reconstruction (ASR) [42]. Data were re-referenced to average reference. Adaptive mixture independent component analysis (AMICA) [43] was applied to the preprocessed scalp-channel data matrix to decompose the channel signals into linear mixtures of the same number of temporally maximally independent component (IC) signals. Locations of the ICs were estimated using equivalent current dipole fitting functions provided by FieldTrip [44]. EEG channel locations were co-registered to the Montreal Neurological Institute (MNI) canonical head model template ‘Colin27’.

Equivalent dipole locations, orientations, and moments were estimated for all ICs using a two-step approach, first by coarse grid search to give an optimized starting location and second by fine fitting using non-linear optimization to minimize residual variance of the single equivalent dipole model. Independent components whose frequency spectra were incompatible with brain sources (e.g., without a 1/f character, etc.) were visually identified and excluded from component clustering using EEGLAB plugin *std_selectICsByCluster* (documented at *sccn*.*ucsd*.*edu/wiki/Std_selectICsByCluster*). We also rejected ICs with equivalent dipole locations outside the brain as well as ICs with scalp maps whose residual variance from the scalp projection of the best-fit equivalent model dipole was larger than 15%.

As a result, 526/1472 ICs were selected for clustering (mean 22.8 per participant, SD ±8, range 6–37) and submitted for the final analysis. Continuous data were separated into trials from -1 s to 2 s relative to array onsets. A mean of 595 trials per participant (SD ±133, range 302–756) were thus extracted and sorted by condition.

*Independent component clustering for group-level analyses*. Retained ICs for all participants were gathered and separated into 15 clusters using EEGLAB k-means clustering based on equivalent dipole location. Scalp topographies, trial-averaged ERPs, mean power spectral densities, and event-related spectrum perturbations (ERSPs) were computed for each IC. To compute the ERSPs, a Morlet wavelet was used using a linearly varying number of cycles, from 3 at the lowest frequency (3 Hz) to 20 at the highest (125 Hz). The sliding data window length was 1116 ms, and the window step size 9.42 ms (3000 ms– 1116 ms) / (200 steps). Spectral power was computed in 50 frequency bins at log-spaced intervals.

*Statistics*. The factorial design of the experiment involved two factors, distractor Heterogeneity (0°, 12°, 24°, 36°) and target Trial type (target-absent, target-present), producing a 4 x 2 design. For selected component clusters of experimental interest, ERSP results were tested using repeated measures ANOVAs with cluster-level correction (Groppe et al., 2011). First, a repeated measures ANOVA was applied to each ERSP pixel to obtain significant interactions across the 4 x 2 factorial design. Applying a pixel-wise p-value threshold of 0.001 produced (uncorrected) results. For multiple comparison correction, cluster-level correction was used; the uncorrected results were evaluated to determine the distribution of significant pixel cluster size. For each cluster, F-values were summed, forming a cluster mass estimate; these were stored for later comparison. Meanwhile, surrogate statistical testing was performed by permuting data labels across the 4 x 2 design, again generating clusters of quasi-significant pixels using an uncorrected 0.001 < p threshold, and the summed F-value mass of each such quasi-cluster was entered into a surrogate cluster mass distribution. This process was repeated 10,000 times to obtain the surrogate distribution. Finally, the 95^th^ percentile value of the surrogate cluster mass distribution was used to threshold the results for the true significant pixel clusters, and only those larger than the critical value were used in the corrected results.

## Results

### Behavioral data

#### Search accuracy

A two-way ANOVA including the within-subject factors distractor Heterogeneity (0° vs. 12° vs. 24° vs. 36°) and target Trial type (target present vs. target absent) revealed there were significantly fewer correct responses with increasing distractor heterogeneity (M_0° Het_ = 89.3% vs. M_36° Het_ = 80.0%), F(3,63) = 47.47, p < .001, η^2^ = .693 and fewer correct responses in target-present trials (M = 83.9%) than in target-absent trials (M = 88.8%), F(1,21) = 6.11, p = .022, η^2^ = .225, see Fig 1B. There was no interaction between Trial type and distractor Heterogeneity (p = .480).

#### Reaction time

A two-way ANOVA including distractor Heterogeneity (0° vs. 12° vs. 24° vs. 36°) and target Trial type (target present vs. target absent) as the within-subject factors revealed significantly shorter RTs in target-present trials (M = 471 ms) than in target-absent trials (M = 510 ms), F(1,21) = 6.98, p = .015, η^2^ = .249, but not affected by heterogeneity (p = .573). An interaction between target Trial type and distractor Heterogeneity revealed that the RT benefit for target present trials was modulated by heterogeneity, F(3,63) = 3.31, p = .026, η^2^ = .136. Follow-up t-tests showed that RTs were shorter in target-present trials than in target-absent trials for Het0° (ΔM = 39 ms, t(21) = 2.34, p = .029), for Het12° (ΔM = 49 ms, t(21) = 2.63, p = .016), for Het24° (ΔM = 57 ms, t(21) = 2.86, p = .009), but not for Het36° (ΔM = 10 ms, t(21) = 0.65, p = .523).

#### Signal detection measures

One-way ANOVAs with within-subject factor distractor Heterogeneity (0° vs. 12° vs. 24° vs. 36°) were conducted for sensitivity measure d-prime and response criterion measure ln(β) (natural logarithm of β). Results showed that d-prime values decreased with increasing distractor Heterogeneity (M_0° Het_ = 3.1 vs. M_12° Het_ = 3.2 vs. M_24° Het_ = 2.7 vs. M_36° Het_ = 2.0), F(3,63) = 43.89, p < .001, η^2^ = .676; there were no effects for β (p = .256).

### ERP data

#### N2pc

The N2pc was significantly different from zero for all heterogeneities (all p < .001) and decreased with heterogeneity (M_het0_ = -2.10 μV, M_het12_ = -1.79 μV, M_het24_ = -1.47 μV, M_het36_ = -1.45 μV), F(3,63) = 5.11, p = .017, η^2^ = .196, see Fig 1C and 1D. Contrasts revealed a linear trend, F81,21) = 15.00, p = .001, η^2^ = .417, but no quadratic or cubic trends (p > .377).

#### Bilateral N2

N2 amplitude decreased with increasing heterogeneity (M_het0_ = -1.41 μV, M_het12_ = -1.28 μV, M_het24_ = -1.00 μV, M_het36_ = -0.85 μV), F(3,63) = 3.33, p = .042, η^2^ = .137, see Fig 1E and 1F. Contrast analyses confirmed a linear trend, F(1,21) = 8.53, p = .008, η^2^ = .289, but no quadratic or cubic trend (p > .648). N2 amplitude was generally larger for target-absent (M = -1.37 μV) than for target-present trials (M = -0.90), F(1,21) = 9.66, p = .005, η^2^ = .315. A two-way interaction of Heterogeneity and Trial-type showed that the target-absent related negativity varied by heterogeneity (ΔM_het0_ = -0.73 μV, ΔM_het12_ = -0.38 μV, ΔM_het24_ = 0.02 μV, ΔM_het36_ = -0.85 μV), F(3,63) = 2.91, p = .041, η^2^ = .122. Follow-up analyses revealed that N2 amplitude differed between target-present and target-absent trials for lowest (t(21) = 3.00, p = .007) and highest heterogeneity (t(21) = 4.28, p < .001) but not for intermediate heterogeneities (p > .160). Contrast analyses confirmed a quadratic trend, F(1,21) = 7.62, p = .012, η^2^ = .266, but no linear or cubic trend (p > .263).

Visual inspection of the waveforms showed that the difference between target-absent and target-present trials continued after the N2 time window into the P3 time window. To further analyze this, we ran the same analysis we used for the N2 for the P3, with the time window of 300–400 ms. This revealed a main effect of Trial type (M_Tabsent_ = -0.21 μV vs. M_Tpresent_ = 2.26), F(1,21) = 44.57, p < .001, η^2^ = .680. There was no effect of heterogeneity (p = .893) and no interaction of Trial type and Heterogeneity (p = .190).

### Group-level analyses on independent component clusters

**Fig 2:**
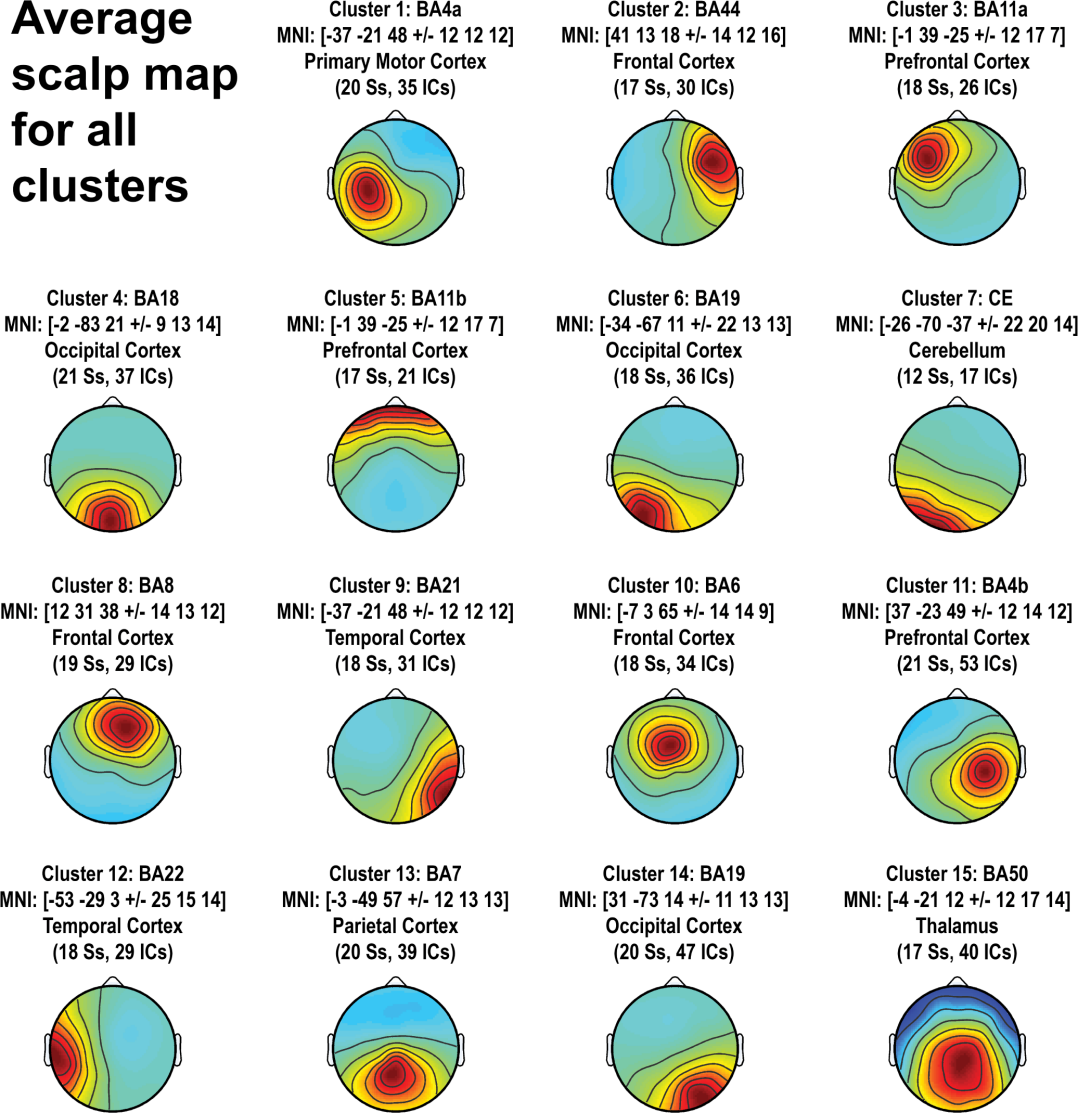
Cluster mean scalp maps. Cluster mean scalp maps are shown with MNI coordinates and respective brain area (BA = Brodman area). Clusters are sorted according to explained variance. In brackets: number of subjects (Ss) contributing to the cluster and total number of independent components (ICs) included in the cluster.

The finally selected 526 ICs from 22 participants were separated into 15 clusters using their equivalent current dipole locations as a criterion. The mean scalp topography of each cluster is shown in Fig 2. The subsequent group-level analyses were applied to a subset of clusters of interest.

#### Significant theta-band interaction in ERSPs for IC Cluster 4

**Fig 3:**
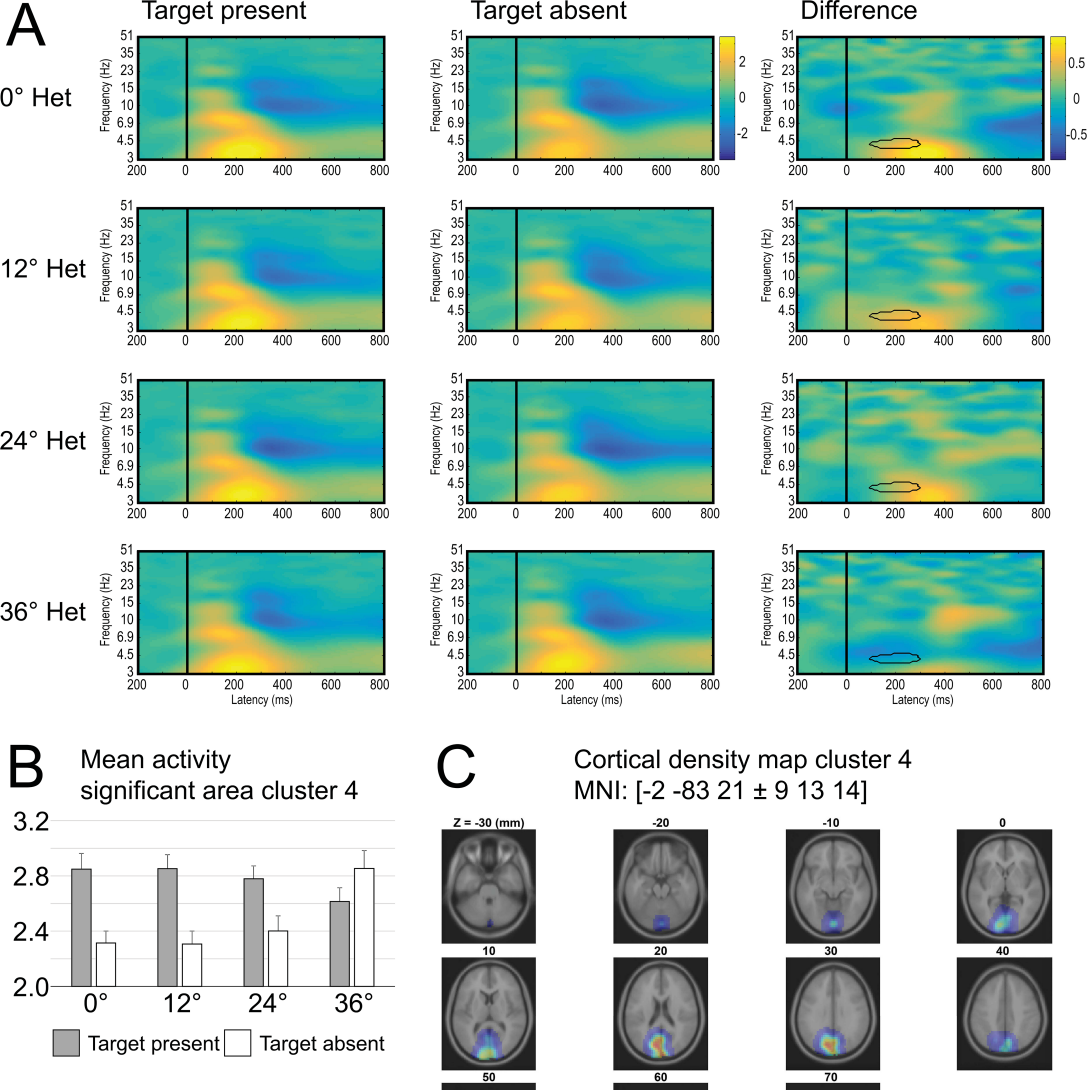
Cluster 4. (A) Time-frequency plot for all levels of heterogeneity (rows) and target-present (left column), target-absent (middle column) trials and the difference between target-present and–absent trials (right column). (B) Bar graphs indicate mean power for area with significant interaction of trial type and level of heterogeneity (highlighted with black outline in difference time-frequency plot). (C) Cortical density maps.

The 2 x 4 ANOVA on distractor Heterogeneity and target Trial type revealed a significant interaction in an ERSP region centered at 5 Hz and 200 (±100) ms following array presentations. The results are shown in Fig 3A. To identify the nature of the interaction, mean ERSP values within the significant ERSP pixel cluster were visualized as a bar graph in Fig 3B. Fig 3 suggests that the effect of Trial Type was stronger under the low distractor Heterogeneity condition, and it decreases as Heterogeneity increases. The ERSP ANOVA for IC Cluster 4 also found a main effect of target Trial Type, with a larger power increase near 4 Hz and 170 ms to 400 ms in the target-present (M = 2.84 dB) than in the target-absent condition (M = 2.41 dB).

#### Significant main effect of target Trial type in the ERSP theta band for IC Cluster 6

**Fig 4:**
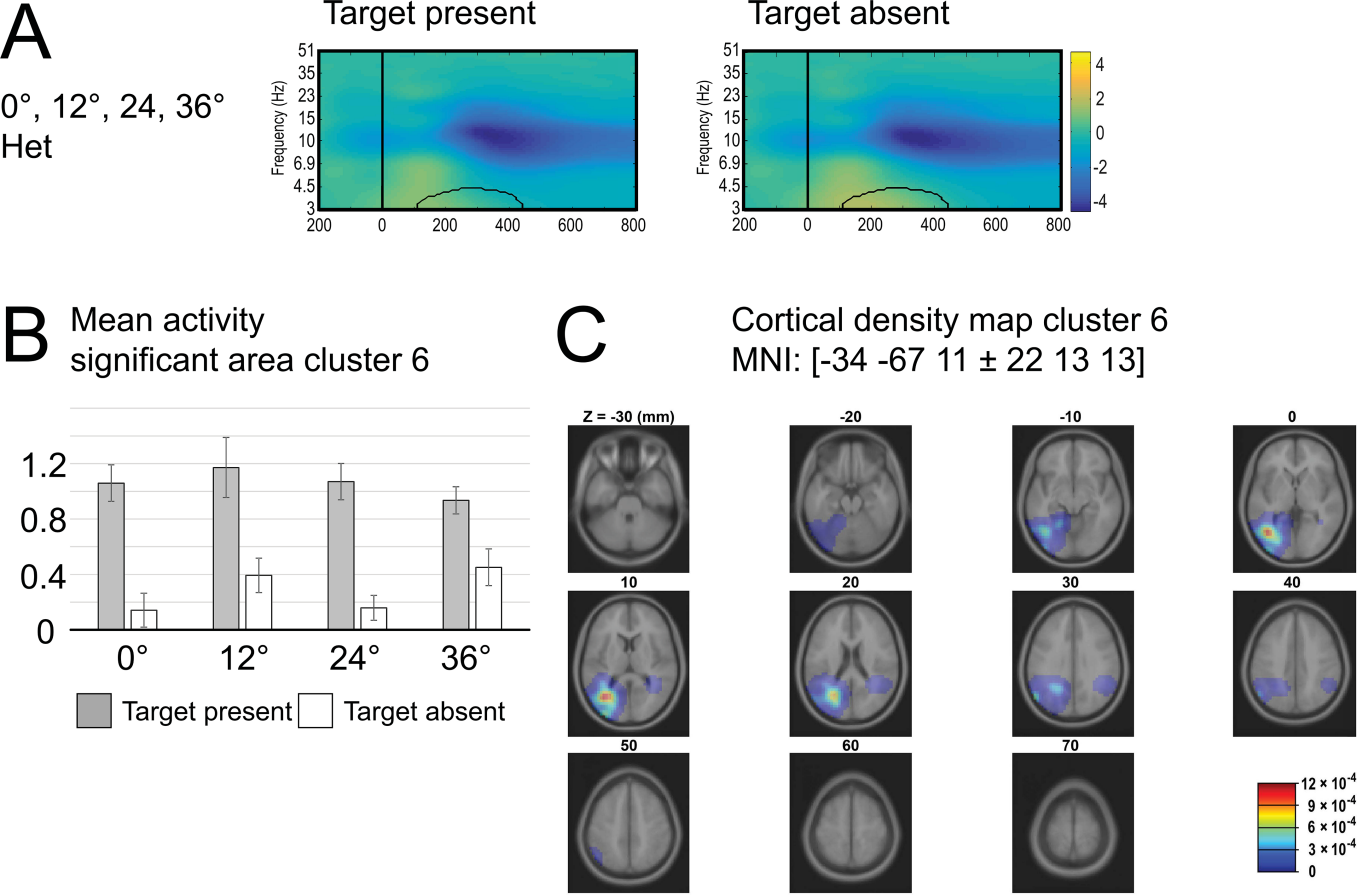
Cluster 6. Time-frequency plot for target-present (left panel) and target-absent (right panel) trials, collapsed across all levels of heterogeneity. Bar graphs indicate mean power for area with significant main effect of trial type (highlighted with black outline in each plot). (C) Cortical density maps.

The analysis revealed the main effect of Trial Type with an increase in theta power around 100 ms– 450 ms in target present condition (M = 1.06 dB) compared with target absent condition (M = 0.29 dB), see Fig 4.

#### Significant main effect of Trial Type and heterogeneity in the ERSP theta band for IC Cluster 14

**Fig 5:**
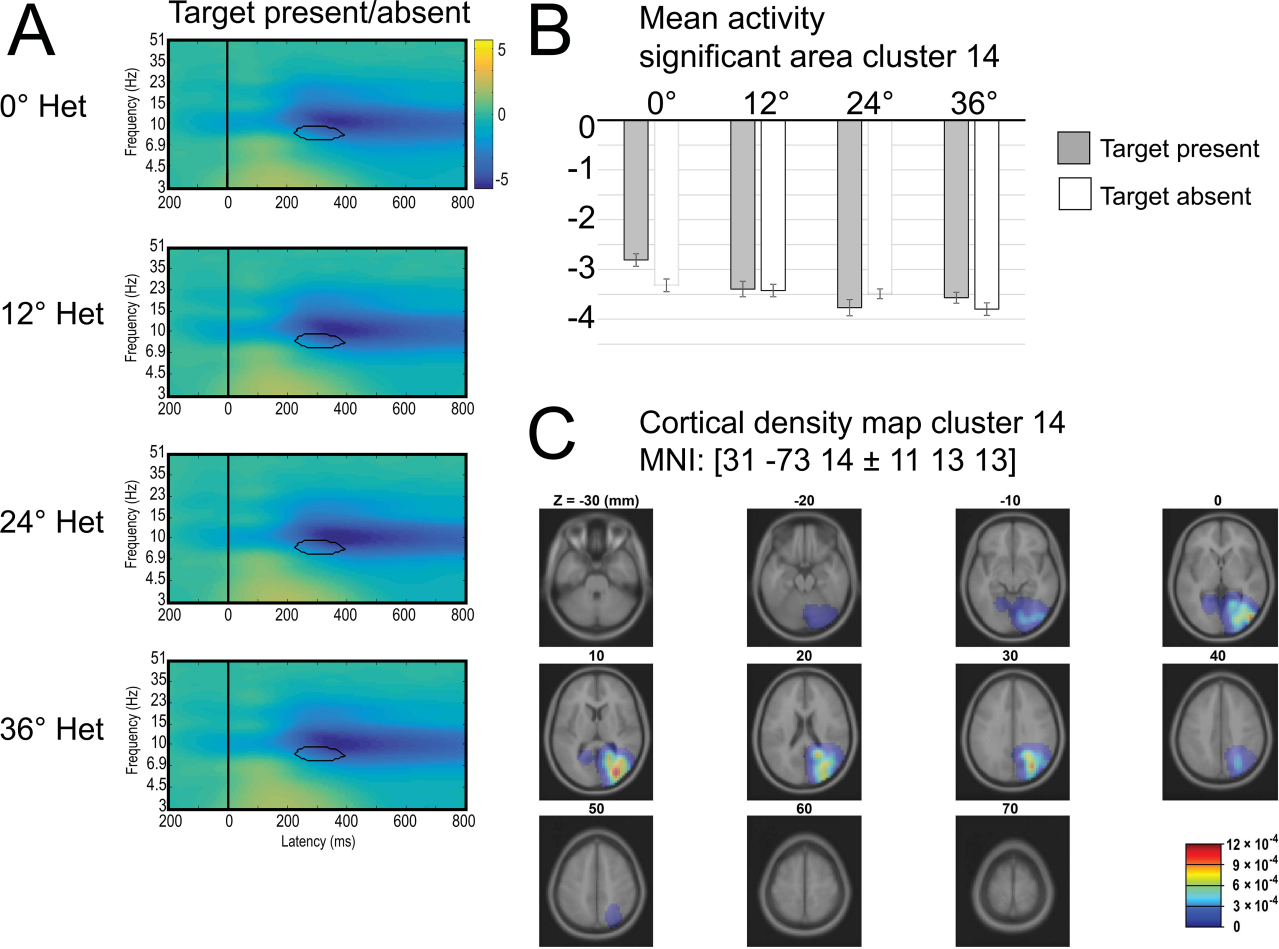
Cluster 14. Time-frequency plot for all levels of heterogeneity, collapsed across target-present and target-absent trials. Bar graphs indicate mean power for area with significant main effect of heterogeneity (highlighted with black outline in each plot). (C) Cortical density maps.

A significant main effect of distractor Heterogeneity on the ERSP was found for IC Cluster 14. The presentation-induced alpha power decrease (alpha blocking) became larger with increasing distractor Heterogeneity (0° ΔM = -3.06 dB; 12° ΔM = -3.4 dB; 24° ΔM = -3.62 dB; 36° ΔM = -3.68 dB;), see Fig 5. Moreover, a main effect of Trial Type was significant in theta band power during 250 ms to 420 ms window. in the target-present condition the (M = 1.33 dB) compared with the target-absent condition (M = 0.68 dB).

## Discussion

In a visual search task, participants had to find a target that was embedded in contexts of varying whole-field heterogeneity. Distractors varied within 0° (all identical), 12°, 24° or 36°. The key finding of the present study was that increasing whole-field heterogeneity resulted in worse search performance due to less efficient attention deployment. Behavioral results showed that accuracy and target sensitivity (d-prime) monotonically declined with increasing context heterogeneity. This is a replication of previous studies [23–26] and shows that searching for a target becomes more difficult when distractors are less similar. Crucially, by recording EEG we could investigate if this behavioral advantage was due to differential attention deployment. N2pc amplitudes monotonically decreased with heterogeneity suggesting that the performance decline is related to less pronounced attention deployment towards targets in more heterogeneous contexts. This is in line with previous studies that measured the N2pc in contexts of varying arrangement heterogeneity [16,17]. For example, when a target is embedded in displays of two groups of identical distractors, they elicit a larger N2pc than a target embedded in displays in which the same distractors are randomly distributed across the visual field [16]. Our study thus extends previous studies and shows that attention deployment is also affected by whole-field heterogeneity. Note that the differences we found in the N2pc cannot be ascribed low-level physical differences because the N2pc is a difference wave between contra- and ipsilateral activity which averages out any low-level physical differences.

The differential bilateral N2 (larger negativity for target-absent trials than target-present trials) that is related to global vs. local processing [11,16,19,45] was replicated in this study. The differential bilateral N2 decreased with increasing heterogeneity up to a heterogeneity of 24°. Surprisingly, however, N2 then increased again for a heterogeneity of 36°. Although this is slightly speculative, this may indicate that least and most heterogeneous contexts are processed most globally, whereas intermediately heterogeneous contexts are processed more locally. When all distractors were identical (0° heterogeneity), it is likely that contexts were processed on a more global level because of strong grouping of distractors into one perceptual unit [3,17,46]. With increasing heterogeneity, the probability that nearby stimuli are similar to one another decreases which in turn decreases the overall number of clusters of grouped items. Based on the finding that the differential bilateral N2 is at the same level for 36° as it was for 0°, one may argue that at the highest level of heterogeneity, grouping is not strong enough anymore to allow processing of local clusters and observers may perceive contexts on a more global level. Alternatively, the bilateral N2 may not reflect global processing. Either way, due to the decoupled pattern of N2pc and bilateral N2 results, it seems that global vs. local processing cannot explain the entire pattern of differential attention deployment. As expected, the P3 also showed a strong difference between target-absent and target-present trials, but no modulation by heterogeneity. P3 amplitude differences due to target presence are a well-established effect and probably related to response preparation [47]. Our results suggest that starting at about 300 ms visual processing as target features are already extracted due to context heterogeneity no longer affect.

So what is the reason for the differential attention deployment in contexts of varying heterogeneity? Since there was no clear border between predefined regions in the visual field, the limiting factor in attention deployment was presumably not the number of perceptual groups, but rather the availability of saliency signals. In the 0° heterogeneity condition, iso-orientation suppression of distractors is strong because they are relatively similar [48–50]. This will increase target representation on the saliency map [7] based on which attention is deployed [51,52]. Increasing heterogeneity will decrease iso-orientation suppression of distractors and increase the activity of their representation on a saliency map, which changes the signal-to-noise ratio. Increasing context heterogeneity decreases target saliency and increases distractor saliency, i.e., the signal-to-noise ratio deteriorates and makes visual search for the target less efficient [53]. The availability of saliency signals may also lead to different processing modes. In contexts with no or almost no heterogeneity, observers can decide at the level of the saliency map if a target is present or not: if a single peak manifests on the saliency map, it clearly indicates target presence and a local attentional focus is not required. If no such peak is present, this clearly indicates target absence. In very heterogeneous contexts, local contrasts lead to high saliency and an increased noise level. Although the initial percept of such heterogeneous contexts may be global due to a breakdown of local grouping, as indicated by the large bilateral N2, a comparison on a global level is not expedient anymore: target-present and target-absent trials do not show globally distinct activities on the saliency maps. Local processing is required which is a time-consuming process. As a result searching in more heterogeneous contexts may be serial, whereas searching in less heterogeneous contexts may be parallel [45].

The ERP data in the present study did not allow us to find neural markers of the differential activation on the saliency map itself, but only of the differential attention deployment based on it (the N2pc). However, the Independent Component Analysis (ICA) used in the present study allowed a more accurate dissection of the highly mixed and blurred channel data into spatially distinct effective source clusters. The regional localizations of these clusters suggest that different neural substrates are involved in the more efficient detection of targets in less heterogeneous contexts. The present study identified three statistically independent sources that were sensitive to changes in target presence/absence and context heterogeneity and may relate to activations on a saliency map.

Most importantly a cluster was identified that showed an interaction between target presence/absence and context heterogeneity in the theta frequency range (4–5 Hz) between 100 and 300 ms after search onset (cluster 4). This cluster showed an increase in theta power in target-absent trials and a decrease in theta power in target-present trials with increasing heterogeneity. This exactly matches the presumed activation on the saliency map: more activity for distractors and less activity for targets with increasing context heterogeneity. The short latency of the observed statistical differences (starting around 150 ms post-stimulus) is also in line with the assumption that activation patterns on a saliency map emerge relatively early. The differential detection difficulty we observed in the behavioral data can be directly derived from the activation pattern of cluster 4: neural states in target-present and target-absent states are more similar in heterogeneous contexts, making it more difficult for the neural system to distinguish between target-present and target-absent trials. As a result, the decision that a target is present or absent was more often incorrect. Dipole localization indicated that this cluster may reflect activity in the cuneus (MNI: x = -2, y = -84, z = 23). In line with this, imaging studies have previously found increased activity in the cuneus for more salient singleton than for less salient non-singleton targets in visual search tasks [54] and for presence versus absence of salient additional singletons [55]. That this cluster showed significant differences in the theta band is in line with the notion that selection based on a saliency map may happen in theta range iterations [56].

How does the visual system deal with the worse signal-to-noise ratio and increased distractor saliency in more heterogeneous contexts? An answer to this question may be provided by activity patterns observed in another statistically significant cluster we identified (cluster 14) in the middle occipital gyrus (MNI: 30, -73, 15), a region that was found to be involved in covert spatial attention [57,58]. This cluster showed slightly later differences than cluster 4, starting around 200 ms, between different levels of heterogeneity in the alpha frequency range. Alpha was generally more negative compared to baseline in this cluster, showing a typical pattern of alpha suppression that was previously observed in tasks requiring selective attention and is usually conceptualized as a gating mechanism: increased alpha power reflects suppression and decreased alpha power reflects enhancement of incoming visual information [33,59–62]. According to the inhibition notion, differential alpha modulation reflects a ‘spotlight of attention’: neural representations of task-relevant areas show less alpha power (= suppression), allowing more efficient processing of incoming information. Neural representations of irrelevant areas, however, show more alpha power which makes stimuli in these areas less likely to be processed. In the present study, alpha power decreased in cluster 14 with increasing heterogeneity, suggesting that more target enhancement and more filtering were required with increasing context heterogeneity.

Finally, cluster 6 showed early differences, starting around 150 ms, between target-present and target-absent trials in the low theta/high delta frequency range (3–4.5 Hz). Power in these low frequencies was more pronounced in target-present than in target-absent trials, which suggests that we identified a cluster that reflects the presence of relevant information. This is in line with the finding that theta band activity is related to attentional sampling of visual stimuli [63,64]. The increased theta/delta power in this cluster might reflect the sampling of the target in target-present trials compared to no such sampling in target-absent trials. Related to this, we found that low theta band activity (4–6 Hz) underlies the N2pc that reflects attention deployment towards task-relevant items [65]. Cluster 6 was localized in the left temporal lobe in the present study (MNI: -35, -67, 10).

In sum, the ICA results show that statistically distinct neural sources contributed to the differential behavioral pattern for targets in contexts of varying heterogeneity. Cluster 4, reflecting activation on the priority map, showed a neural pattern in the theta band that distinguished more (non-heterogeneous) or less (heterogeneous) between target-present and target-absent trials. More (heterogeneous) alpha suppression is then observed in cluster 14 to resolve the ambiguity in cluster 4. This finally leads to a clearly distinct neural pattern between target-present and target-absent trials in cluster 6 based on which observers can decide whether a target was present or absent. As it stands, the ICA results dovetail with the ERP and behavioral results. However, since the ICA approach is exploratory at this point, our findings need to be considered carefully and future research will have to provide converging evidence by using an ICA approach in a larger variety of search tasks.

### Conclusions

In a visual search task, simple line elements serving as distractors could vary by 0°, 12°, 24°, or 36° within a display, inducing different levels of whole-field heterogeneity. Observers had to detect a line orthogonal to the background which was increasingly difficult with increasing heterogeneity. N2pc results suggest increased heterogeneity made the task more difficult because of participants were less able to efficiently deploy their attention towards the targets with increasing heterogeneity. The bilateral N2 that is typically indicative of local/global processing and grouping processes could not provide any evidence for why attention deployment was different in contexts of different heterogeneity. Independent component analyses (ICAs), however, complemented N2pc results nicely and showed that at least three statistically different neural sources contributed to the differential behavioral performance. One reflected activity on the saliency map, one reflected how much filtering was required and one reflected target presence or absence.
